# Chiral packings in cylinders are ultrasensitive to confinement deformation

**DOI:** 10.1038/s41467-026-74709-2

**Published:** 2026-07-16

**Authors:** Xuebin Wang, Jiahao Guo, Yao Li

**Affiliations:** School of Physics and Key Laboratory of Functional Polymer Materials of Ministry of Education, Nankai University, and Collaborative Innovation Center of Chemical Science and Engineering, Tianjin, China

**Keywords:** Statistical physics, thermodynamics and nonlinear dynamics, Biophysics, Applied mathematics

## Abstract

Sphere packings in circular cylinders have attracted substantial research interest, among which the discovery of chiral helical structures is the most iconic. However, recent experimental results on zebrafish do not match the known packing structures in circular cylinders. To account for the inherent imperfections of biological tubes, we take elliptic cylinders as the canonical deformation of circular cylinders and investigate the densest packings of hard spheres in them using simulation, theory, and experiments. Starting from the chiral structures in circular cylinders, we demonstrate that even a weak cross-sectional deformation can trigger entirely new phases, including ones that either eliminate global chirality or significantly complicate the chiral structures. This reveals the significant effect of cylindrical anisotropy. The new helical phases under anisotropic confinement remain chiral and develop hierarchical periodic structures, which are difficult to obtain by simulations but are predicted by our newly developed theory for helical phases in elliptic cylinders. The theory also predicts double oscillated-chain phases without chirality, which perfectly match the simulations. Our work offers fresh insights into understanding packings in anisotropic cylinders, which will help researchers to design new materials and to understand many living systems.

## Abstract (web summary)

Understanding the packing of spheres in confined geometries, particularly in biological systems, is crucial for elucidating the structural and functional properties of various materials. This study reveals that even minor deformations in cylindrical confinement can lead to the emergence of packing phases, including complex helical and achiral double oscillated-chain structures.

## Introduction

The packing problem^1^ originates from Kepler’s conjecture, which has attracted centuries of study by mathematicians and physicists, and Hales eventually completed a proof with computer assistance^2^. It is important to understand how the properties of soft matter systems depend on the spatial distribution of constituents, which relates to the particle packing. Additionally, studies of both ordered periodic arrangements and disordered arrangements often invoke sphere packings. The packings of particles provide insights into the structures and self-assembly properties of condensed matter systems, and have been extensively investigated in mathematics, physics, biology, computer science, and other related fields^3–5^.

In recent years, the densest packings in confined systems have garnered significant attention, with notable progress achieved in cylindrical confinement. Such structures are commonly termed columnar crystals. For the densest packings of hard spheres in circular cylinders^6–15^, when the diameter ratio satisfies $D/d\in \left(1,2.7013\right)$, all spheres are in contact with the cylinder wall, and various chiral helical structures emerge. In addition, investigations of soft spheres confined in circular cylinders have also observed many chiral helical structures, which vary as the pressure is adjusted^16–19^. These columnar crystal structures have been observed in various systems, such as foams^20–22^, colloids^23–29^, and polymers^30–32^, and have had many applications^33–35^. The densest packings of nonspherical particles, such as ellipsoids^36,37^ and nanodumbbells^38^ confined within circular cylinders, have been investigated, revealing new structures.

While packings in ideal cylinders are elegant and receive great successes, real-world tubes—especially in biological systems—are often imperfect. Recent studies^39–42^ have demonstrated that the cellular packing structures in the zebrafish notochord have no counterparts among packings in circular cylinders, suggesting that the notochord is anisotropic. However, how non-circular cylindrical confinement tunes the densest packings remains unclear. Here, we examine the packings of hard spheres in elliptic cylinders to explore the effects of cylindrical anisotropy on the densest packing structures.

In this article, we study the densest packings of hard spheres confined in elliptic cylinders, focusing on the small cylinder anisotropy regime. Remarkably, we find that vanishingly small anisotropies are enough to trigger the emergence of completely new phases. Helical phases are tuned when compared with those observed in circular cylinders, whereas double oscillated-chain phases are absent in circular cylinders. We develop a theory for helical phases in elliptic cylinders that, for sufficiently large sphere numbers, approaches the ideal densest packings. However, because hierarchical periodicity is disrupted at small sphere numbers, the theoretical densest packings differ from the simulation results. This theory is also employed for double oscillated-chain phases, which yield results consistent with the simulations. All phases predicted by our simulation and theory are found in our macroscopic experiments on confined packings of table-tennis balls. Our results encourage further investigations into self-assembly processes in biological systems featuring elliptic cylindrical structures.

## Results

### Phase diagram

**Fig. 1:**
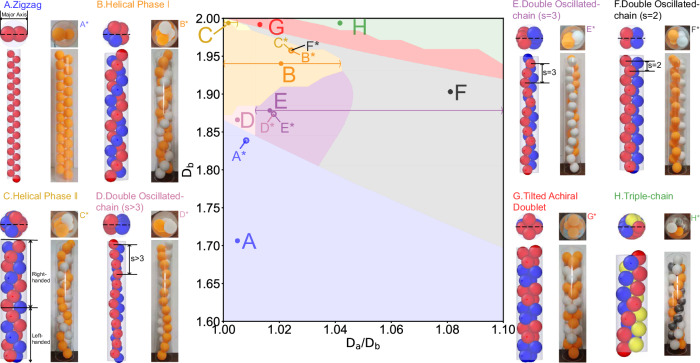
Phase diagram. Simulations of elliptic cylinders with minor axis ${D}_{b}\in \left[1.6,2\right]$, aspect ratio ${D}_{a}/{D}_{b}\in \left[1,1.10\right]$, and sphere numbers *N *≤ 25 are performed. Here, the sphere diameter is taken as the unit length, i.e., *d* = 1, and all other quantities are expressed relative to *d*. The phase diagram contains eight regions of densest packing structures (*A* − *H*), with representative snapshots displayed beside it. Each snapshot is obtained at the parameters of its respective solid point in the phase diagram. The corresponding phase structures observed in the macroscopic experiments of table-tennis ball packings (A^*^ − H^*^) are shown beside the snapshots, which are obtained at the parameter values indicated by hollow points in the phase diagram (The points ${G}^{*}\approx \left(1.0086,2.03\right)$ and ${H}^{*}\approx \left(1.0072,2.08\right)$ are not marked). The spheres are colored differently in the snapshots and the experimental figures, which helps to better illustrate the structures. The three segments in the phase diagram correspond to the value ranges of the three subfigures in Fig. 3.

We investigate the densest packings of hard spheres in elliptic cylinders with minor axis ${D}_{b}\in \left[1.60,2.00\right]$ and aspect ratio ${D}_{a}/{D}_{b}\in \left[1.00,1.10\right]$. When *D*_*a*_/*D*_*b*_ = 1.00, the cylinders have circular cross-sections, so for *D*_*a*_/*D*_*b *_≤ 1.10, the elliptic cylinders are obtained by slight deformations of the circular cylinders. Through Monte Carlo (MC) simulations, we find that even slight elliptic deformations lead to the emergence of various densest packing structures (Fig. 1): zigzag, helical phases, double oscillated-chain phases, tilted achiral doublet, and triple-chain. The most interesting results in this study are the first two findings: the double oscillated-chain phases and the helical phases. These two phases evolve from the known chiral helical phase observed in circular cylinders.

The double oscillated-chain configurations consist of two identical chains with oscillated segments, which can be further classified by the segment length *s*: *s* = 2 (Fig. 1F), *s* = 3 (Fig. 1E), and *s* > 3 (Fig. 1D). These structures are novel phases that are not observed in circular cylinders, and they match well the packing structures of vacuolated cells in the zebrafish notochord. They emerge when the cylinder is even slightly deformed, indicating that the packings are highly sensitive to small changes in cylinder shape. Therefore, they play an important role in packing in slightly elliptic cylinders, which we will discuss in detail later.

The helical phases evolve from the single/double helix structures in circular cylinders and can be viewed as two helical chains intertwined with each other. In the simulation, we observed two types of helical phases: Helix phase I is monochiral, without any defects (Fig. 1B); helix phase II is bichiral, with two defects that divide the structure into two segments of opposite chirality (Fig. 1C). However, due to limitations on the number of spheres, the densest helical packings cannot be reliably obtained by simulations, but we address this issue by developing a new theory.

The latter three phases: the zigzag structure is identical to that observed in circular cylinders (Fig. 1A); the tilted achiral doublet in elliptic cylinders is related to the achiral doublet in circular cylinders, and its basic unit is formed by four spheres arranged in a cross-shaped pattern (Fig. 1G); the triple-chain structure consists of three chains, in which spheres within each chain are not necessarily in contact and do not form a helical structure (Fig. 1H). We also perform macroscopic experiments in which table-tennis balls (with diameter  ~ 40mm) are packed into transparent elliptic tubes, serving as a complement to our predictions. All phases predicted by the simulations and theory are successfully identified in the experiments by multiple packing trials with tubes of different sizes (Fig. 1A^*^ − H^*^). Further experimental details are given in Section G of the Supplemental Materials See supplemental material.

### Zigzag phase

First, we discuss the simplest zigzag structure. For a circular cylinder with the diameter equal to the sphere diameter (*D* = *d*), spheres form a straight chain. When *D* increases slightly beyond *d*, the available space inside remains limited. Adjacent spheres move apart to reduce vertical height differences, thereby maximizing space utilization and forming a zigzag structure. Unlike circular cylinders, elliptic cylinders provide more space along the major axis. Therefore, the zigzag in elliptic cylinders strictly aligns with the major axis, whereas in circular cylinders it can form along any diametrical direction (details in Section A of the Supplemental Material See supplemental material).

### Helical phases

To better explain the densest packing structures, we develop a theoretical numerical method (see Methods). For a given number of spheres, we can construct the packing structure using this method. Theoretically, as the sphere number approaches infinity, this method yields the densest structure; however, since only a finite number of spheres can be used in calculations, periodic boundary conditions must be applied when employing this method. In this way, when the sphere number *N*≤*N*_*max*_ (where *N*_*max*_ is a positive integer), we can find the structure with the maximum packing fraction among these cases (the corresponding sphere number is the optimal number of spheres N^*^). When *N*_*max*_ is relatively small, we can calculate the results under a finite sphere number and validate them against simulations. As *N*_*max*_ increases, we can approximately find the period of the structure and obtain the densest helical packing. Further details are provided in Section C of the Supplemental Material See supplemental material.

**Fig. 2:**
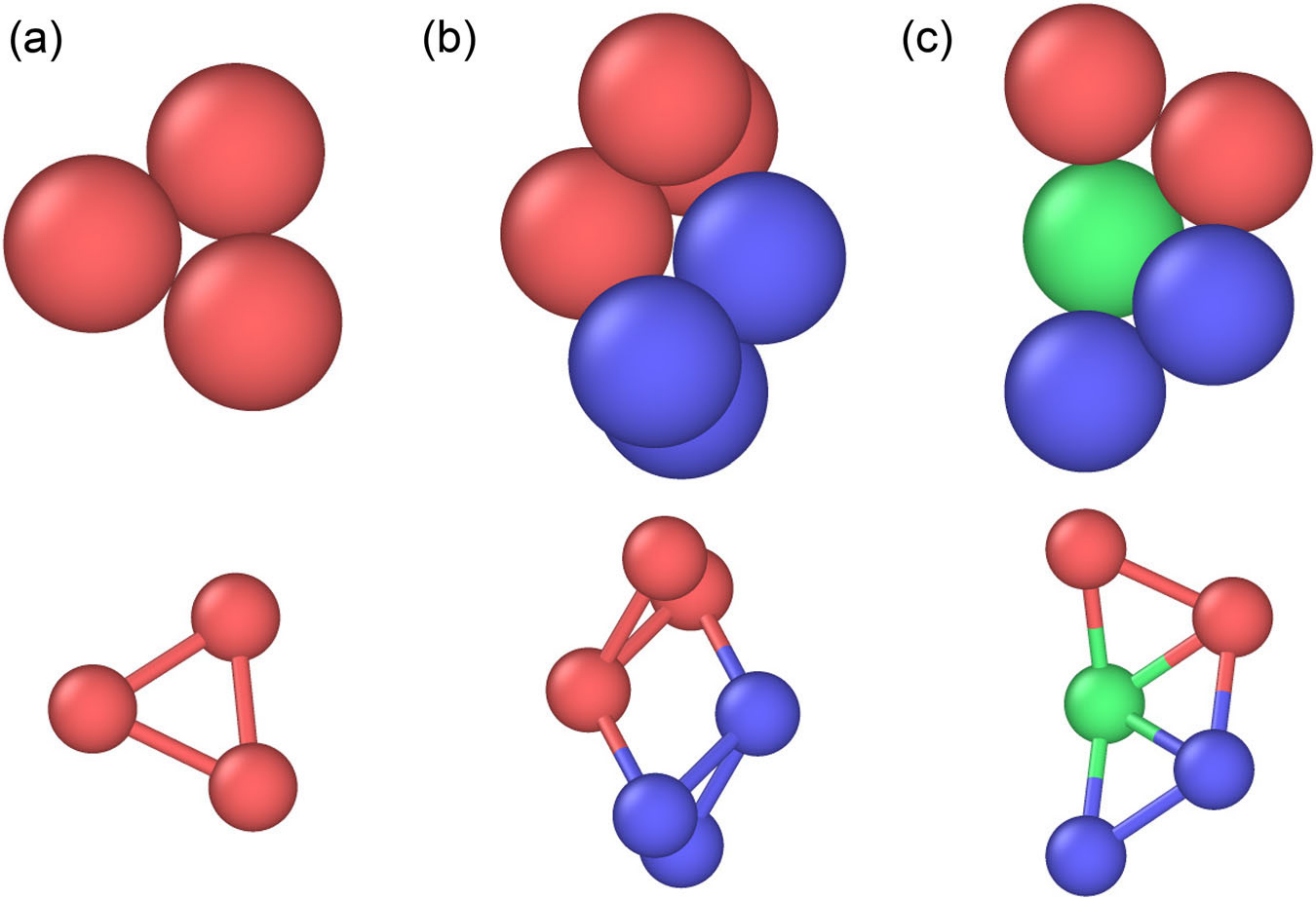
Typical blocks in packing structures. The first row shows snapshots of the actual spheres, and the bonds in the second row indicate the contact relationships between spheres. **a** A triplet of spheres comprises three spheres in contact with each other and with the wall, exhibiting chirality. It appears in various structures and is used in the triplet-sphere packing block method. **b** The central red and blue spheres have an angular difference of *π* and do not contact each other, forming a defect that appears in helical phase II. Triplets composed of red and blue spheres exhibit opposite chiralities. **c** Red-green and blue-green triplets display opposite chiralities, a feature that appears in double oscillated-chain phases.

**Fig. 3:**
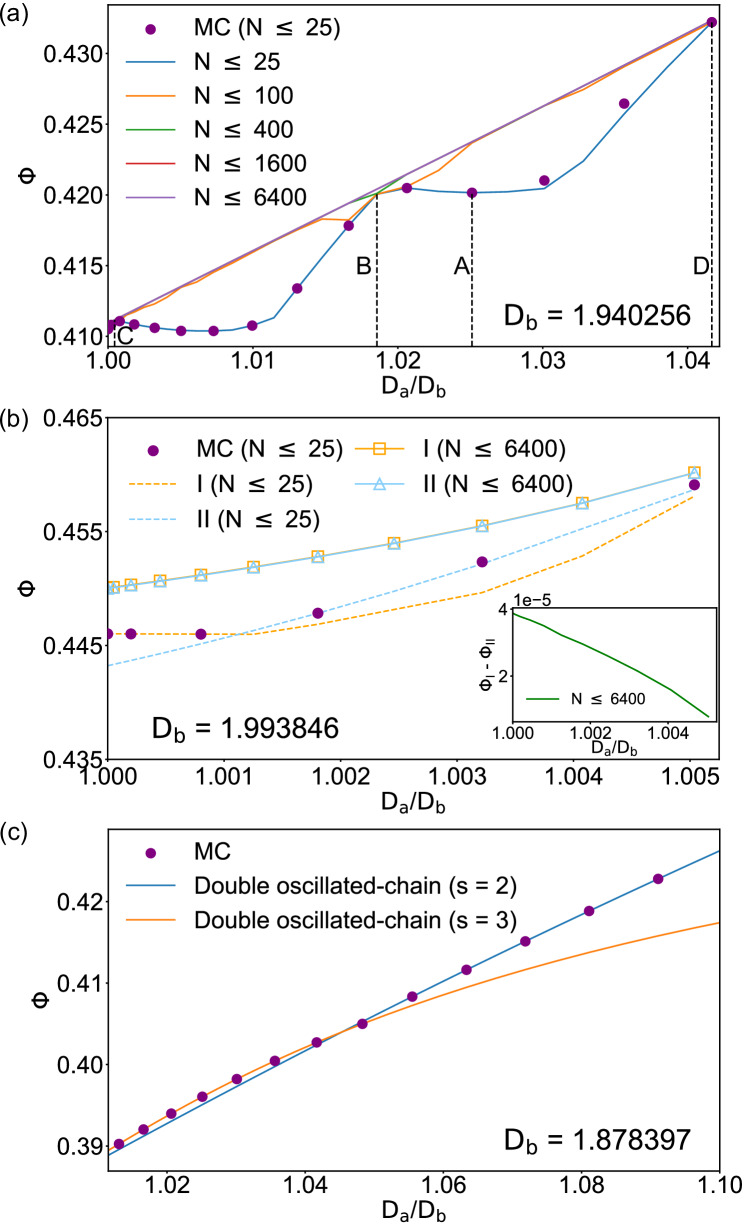
Packing fractions. The packing fractions *ϕ* from theoretical predictions (lines) are compared with the packing fraction *ϕ* from *N *≤ 25 MC simulations (purple points). **a** For *D*_*b*_ = 1.940256, densest structures are derived using the triplet-sphere packing block method at *N *≤ 25, 100, 400, 1600, 6400. Here, the curves for *N *≤ 1600 and *N *≤ 6400 nearly coincide. Structures corresponding to the dashed lines A, B, C, and D are presented in Fig. 5a–d, respectively. **b** For *D*_*b*_ = 1.993846, densest structures are found using the triplet-sphere packing block method (helical phase I) and the defective triplet-sphere packing block method (helical phase II) at small (*N *≤ 25) and large (*N *≤ 6400) sphere numbers. For large sphere numbers (*N*≤6400), both methods yield nearly identical packing fractions, but the small inset at the lower right indicates that the triplet-sphere packing block method achieves a slightly higher *ϕ*. **c** For *D*_*b*_ = 1.878397, the theoretical results for the double oscillated-chain (*s* = 2 and *s* = 3) perfectly match the simulation data and the transition between phase structures.

Helical phase I is formed by triplets of spheres [shown in Fig. 2(a)] with identical chirality. For adjacent spheres labeled *n* and *n* + 1 in the structure, the vertical height difference between them is denoted by Δ*z*_*n*,*n*+1_. In circular cylinders, the single and double helices can be characterized by one and two values of Δ*z*_*n*,*n*+1_, respectively^15^. However, for helical phase I in elliptic cylinders, Δ*z*_*n*,*n*+1_ takes multiple values (more than two), indicating that the structure is composed of various distinct triplets of spheres and can be constructed using the triplet-sphere packing block method. Figure 3a shows the packing fraction *ϕ* as a function of aspect ratio *D*_*a*_/*D*_*b*_ when the minor axis *D*_*b*_ = 1.940256 (the orange segment in the phase diagram of Fig. 1). The triplet-sphere packing block method applied for sphere numbers *N*≤25 predicts most of the MC simulations. Extending the method to larger *N* (*N*≤100, 400, 1600, and 6400), the packing fraction *ϕ* increases with *N* and clearly exceeds the simulations. This implies that periodic boundary conditions with finite *N* affect the densest packings. When *N* is small, we may not be able to obtain the structures of the densest helical phase. To satisfy the periodic boundary conditions, several spheres at the boundary do not form a triplet of spheres but instead form defects, resulting in a low packing fraction. As *N* increases, the densest helical structures can possibly be reached. Meanwhile, the defects caused by the periodic boundary conditions may vanish or become less important, so the packing fraction increases significantly. The curves for *N*≤6400 and *N*≤1600 are almost identical, indicating that the ideal densest packing for the infinite *N* system is approached. For *N*≤25, to satisfy the periodic boundary conditions, the triplet-sphere packing block method must impose a large Δ*z*_*N*,1_ = Δ*z*_*N*,*N*+1_, which can be reduced in some cases when the simulated configurations partially break the triplet-sphere structures, thereby yielding a slightly higher *ϕ*.

**Fig. 4:**
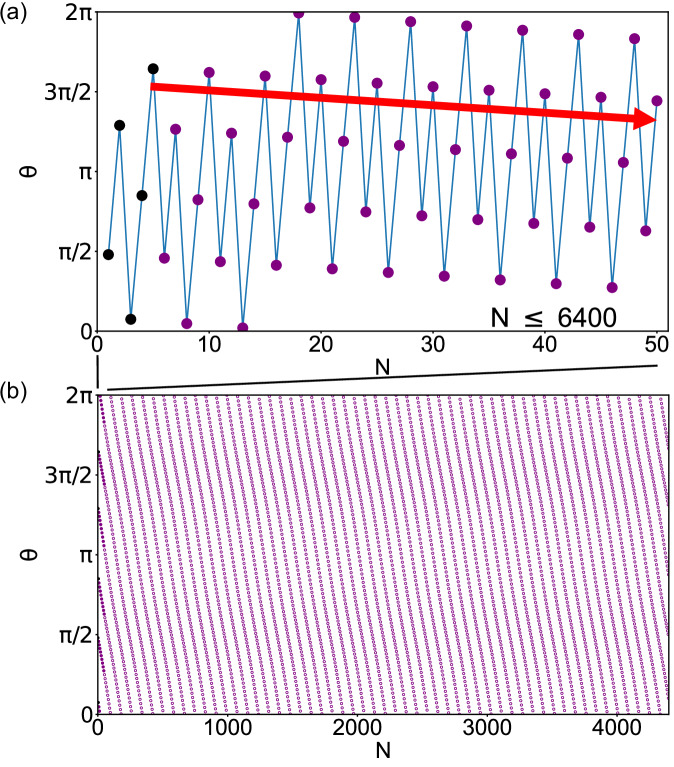
The hierarchical periodic structure. When *D*_*b*_ = 1.940256 and *D*_*a*_/*D*_*b*_ = 1.02512, the optimal number of spheres is N^*^ = 4397 for *N *≤ 6400. Solid dots denote the first 50 spheres, hollow dots denote the following spheres, and black solid dots mark the first subperiod. **a** The angular positions θ of the first 50 spheres are shown, revealing that the structure is composed of five-sphere subperiods. Red arrows indicate the angular offsets of spheres across subperiods. **b** The angular positions θ of all 4397 spheres reveal a longer-period structure.

To further study the helical structures, we observe the angular position of each sphere in the densest packings and discover the hierarchical periodic structures. When *N*≤6400, with minor axis *D*_*b*_ = 1.940256 and aspect ratio *D*_*a*_/*D*_*b*_ = 1.02512, the optimal number of spheres is *N* ^*^ = 4397, and the angular position θ of each sphere is plotted in Fig. 4. Figure 4a shows the angular positions of the first 50 spheres. The red arrow indicates the angular change between spheres separated by four other spheres. The direction of each change is consistent, and the magnitudes of the changes are similar. This suggests that a group of five spheres exhibits an approximate periodicity and nearly completes one full rotation around the inner wall of the elliptic cylinder. However, the rotation angle differs slightly from 2*π*, producing subperiods with offset angular positions. Multiple subperiods combine to form a higher-level period. Figure 4b presents the full period of all 4397 spheres, suggesting that the helical phase forms a higher-level periodic structure composed of nested subperiods.

**Fig. 5:**
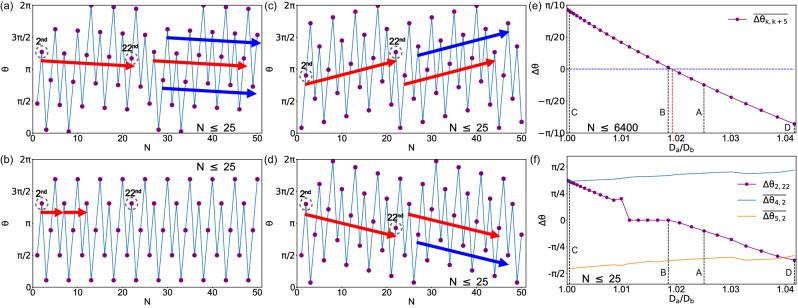
Discussion of the effect of periodic boundary conditions on the structure. For minor axis *D*_*b*_ = 1.940256: for sphere number *N*≤25, we use the triplet-sphere packing block method to find the densest structures, and then apply periodic boundary conditions to obtain the angular positions of the first 50 spheres for four cases. **a** aspect ratio *D*_*a*_/*D*_*b*_ = 1.02512; the optimal number of spheres is N^*^ = 25. **b** *D*_*a*_/*D*_*b*_ = 1.018554, N^*^ = 5. **c** *D*_*a*_/*D*_*b*_ = 1.000450, N^*^ = 22. **d** *D*_*a*_/*D*_*b*_ = 1.041667, N^*^ = 23. The arrows indicate how the angular positions of spheres change across subperiods over one period. In Figure (**a**), the spheres marked by the left red arrow are not collinear with those marked by the three right arrows. In (**b**–**d**), the spheres marked by the left red arrow lie on the same line as the spheres marked by the red arrow **(b**), the upper blue arrow (**c**), and the lower blue arrow (**d**) on the right, respectively. **e** Using the triplet-sphere packing block method to find the theoretical densest packings (*N *≤ 6400), we find that the average-angular offset of the spheres between subperiods, $\overline{\Delta {\theta }_{k,k+5}}$, decreases as the aspect ratio increases, and that when the aspect ratio *D*_*a*_/*D*_*b*_ ≈ 1.019 (marked by the red dashed line, which is near case B), the average-angular offset is zero. **f** For *N *≤ 25, we plot the angular difference Δθ_2,22_ between the 2^nd^ and 22^nd^ spheres of the constructed structures for various aspect ratios, and we further compute the average-angular differences between the 2^nd^ and 4^th^ spheres, $\overline{\Delta {\theta }_{4,2}}$, and between the 2^nd^ and 5^th^ spheres, $\overline{\Delta {\theta }_{5,2}}$, within the subperiods. Case C is located in the region where Δθ_2,22_ approaches $\overline{\Delta {\theta }_{4,2}}$, while case D is located in the region where Δθ_2,22_ approaches $\overline{\Delta {\theta }_{5,2}}$.

In general, the packing fractions of the constructed structures for *N *≤ 25 match the simulation results but differ significantly from the theoretical densest packings. For example, in Fig. 3a, the packing fraction of the constructed structure for *N *≤ 25 represented by dashed line A is obviously lower than that of the theoretical densest structure. The constructed structure represented by dashed line A is given in Fig. 5a. Comparing the structure shown in Fig. 5a with the theoretical helical structure in Fig. 4a reveals that, for *N *≤ 25, the angular positions undergo a sudden change at the period boundary. This indicates that, for structures constructed with a small number of spheres, periodic boundary conditions have a significant influence, thereby preventing the formation of the hierarchical period.

Specifically, for *N*≤25, the constructed structures corresponding to the cases indicated by dashed lines B, C, and D in Fig. 3a have packing fractions approaching the densest packings. Dashed lines B, C, and D represent cases B, C, and D, and the corresponding structures are shown in Fig. 5b–d, respectively. Figure 5e shows that the theoretical densest structure for case B has a vanishing average-angular offset between subperiods $\overline{\Delta {\theta }_{k,k+5}}\triangleq \overline{{\theta }_{k+5}-{\theta }_{k}}\approx 0$. Therefore, for *N*≤25, the subperiod becomes the period, and the constructed structure approximates the densest packing [see Fig. 5b]. In Fig. 5f, we compare the angular difference Δθ_2,22_ ≜ θ_22_ − θ_2_ between the 2^nd^ and 22^nd^ spheres with the average-angular differences $\overline{\Delta {\theta }_{4,2}}\triangleq \overline{{\theta }_{5k+2}-{\theta }_{5k+4}}$ and $\overline{\Delta {\theta }_{5,2}}\triangleq \overline{{\theta }_{5k+2}-{\theta }_{5k+5}}$ (the average-angular differences between the 2^nd^ and 4^th^, and between the 2^nd^ and 5^th^ spheres within each subperiod, respectively). The angular difference Δθ_2,22_ approaches $\overline{\Delta {\theta }_{4,2}}$ near C, and approaches $\overline{\Delta {\theta }_{5,2}}$ near D. The structures for cases C and D are shown in Fig. 5c, d, respectively. In Fig. 5c, the sequence formed by the 2^nd^ sphere within each subperiod of one period is collinear with the sequence formed by the 4^th^ sphere within each subperiod of the subsequent period. Fig. 5d shows a similar collinear relationship between the sequences formed by the 2^nd^ and 5^th^ spheres. Collinearity reduces small-system periodic-boundary effects, enabling the structure to approach the theoretical densest packing fractions. More detailed analysis is given in Section D of the Supplemental Material See supplemental material.

In helical phase II, helical chains invert chirality twice (Fig. 1c), with defects emerging at each inversion point. These defects resemble those introduced in the related work^11,12^. The triplets of spheres above and below the defect exhibit opposite chiralities, and the two spheres at the defect have an angular difference of *π* without contact [Fig. 2b]. The two defects divide the structure into two segments of equal sphere numbers but opposite chiralities, each defect acting as a center of inversion symmetry. By placing one defect at the center, generating the other at the periodic boundary through periodic boundary conditions, and accounting for chirality reversal, the structure can be constructed using the triplet-sphere packing block method. This process is referred to as the defective triplet-sphere packing block method. Fig. 3b shows the case with minor axis *D*_*b*_ = 1.993846 (the yellow segment in the phase diagram of Fig. 1), where increasing the aspect ratio *D*_*a*_/*D*_*b*_ causes the structure to transition from helical phase I to helical phase II. For *N*≤25, structures built via the triplet-sphere packing block method exhibit higher packing fraction *ϕ* at small *D*_*a*_/*D*_*b*_, while the defective triplet-sphere packing block method achieves higher *ϕ* at large *D*_*a*_/*D*_*b*_, which is consistent with the trend observed in the simulation results. However, for the defective triplet-sphere packing block method, the resulting *ϕ* is significantly lower than the simulation results at larger *D*_*a*_/*D*_*b*_. This is because the structure achieves higher packing fraction by partially disrupting some triplets of spheres, which may also be related to periodic boundary conditions and the finite number of spheres. For large sphere numbers [*N *≤ 6400 in Fig. 3b], both construction methods produce nearly identical packing fractions. In fact, the triplet-sphere packing block method yields a slightly higher packing fraction *ϕ*, indicating that helical phase II transitions to helical phase I as *N* increases, and suggesting that helical phase II is specific to small *N*.

### The double oscillated-chain phases

**Fig. 6:**
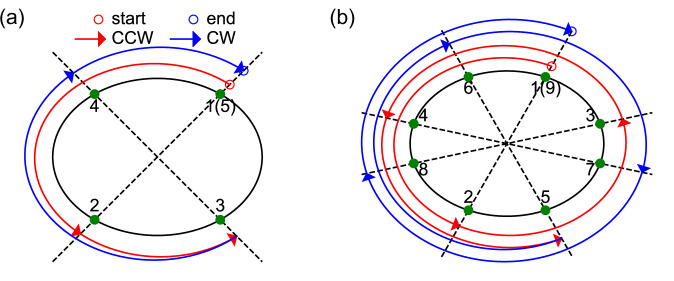
Sketches of the double oscillated-chain for segment lengths *s* = 2 and *s* = 3 (see Fig. 1). Spheres are labeled from bottom to top, and arrows indicate the rotational directions. Red arrows denote counterclockwise rotations, and blue arrows denote clockwise rotations. A single period of (**a**) the double oscillated-chain (*s* = 2) and (**b**) the double oscillated-chain (*s* = 3) is demonstrated. During packing, changes in rotational direction give rise to chirality reversals.

The slight anisotropy even produces the novel double oscillated-chain structure, which also comprises triplets of spheres. However, the chirality alternates between positive and negative, forming a periodic pattern, which makes global structures achiral. Figure 1d–f show the phase structures, while Fig. 2c shows the details of the chirality transition point. To elucidate the packing structures, top-view sketches of the double oscillated-chain for *s* = 2 and *s* = 3 are provided in Fig. 6. In this figure, spheres are packed sequentially from bottom to top and labeled accordingly, and arrows indicate the rotational directions between adjacent spheres. Chirality reversals in the triplets of spheres arise from changes in the rotational direction. The double oscillated-chain (*s* = 2) has a four-sphere periodicity, with all sphere positions symmetric with respect to both the *x*- and *y*-axes. The rotational direction from sphere 2 to sphere 3 is counterclockwise, while that from sphere 4 to sphere 5 is clockwise, resulting in a reversal of chirality. Notably, the angular difference between sphere 1 and sphere 2 is *π*, and that between sphere 3 and sphere 4 is also *π*, which makes no distinction between clockwise and counterclockwise rotations (one choice of rotational direction is shown in Fig. 6). The double oscillated-chain (*s* = 3) has an eight-sphere periodicity, with all sphere positions symmetric with respect to both axes. Analogous analysis also reveals alternating rotational directions during packing. The two structures can still be generated via the triplet-sphere packing block method, provided that the packing permits reversal of rotational directions. Specifically, for *s* = 2, we place four spheres in the cylinder. During the packing process, the rotations from sphere 1 to sphere 2 and from sphere 2 to sphere 3 have the same direction, while the rotation from sphere 3 to sphere 4 is opposite that direction. Similarly, for *s* = 3, the rotation direction from sphere 1 to sphere 5 is opposite to that from sphere 5 to sphere 8. Varying the initial sphere positions yields different packings, and the structure with the maximum packing fraction is chosen as the theoretical result. Owing to the exact periodicity, theoretical predictions match simulation results perfectly, as shown in Fig. 3c (*D*_*b*_ = 1.878397, the purple segment in the phase diagram of Fig. 1). The double oscillated-chain (*s* > 3) follows analogous but more complex patterns, which are not detailed here.

### The other phases

When nearest-neighbor spheres lie at the same height, two adjacent pairs form an intersecting cross structure, yielding the achiral doublet (observed in circular cylinders at *D* = 2). Increasing the minor axis *D*_*b*_ or the aspect ratio *D*_*a*_/*D*_*b*_ transforms the cross structure from horizontal to tilted, with adjacent cross structures exhibiting opposite tilt directions, termed the tilted achiral doublet. The triple-chain structure comprises three distinct chains; however, these chains may include segments where spheres are not in direct contact but still exhibit a tendency to form a chain. Additionally, no perfect helical structure is formed in this case. The tilted achiral doublet and the triple-chain may be influenced by periodic boundary conditions and finite sphere numbers, which will be the focus of our future work.

## Discussion

In summary, we investigated the densest packings of hard spheres confined within near-circular elliptic cylinders. We used simulated annealing to study systems with minor axis ${D}_{b}\in \left[1.6,2\right]$, aspect ratio ${D}_{a}/{D}_{b}\in \left[1,1.10\right]$, and sphere numbers *N*≤25, identifying several distinct phase configurations. Through analysis of triplets of spheres, we elucidated the helical phases and double oscillated-chain phases. The most iconic packings in circular cylinders, the chiral helical structures, are sensitive to the anisotropy of confinement. We observed that the helical phases in elliptic cylinders consist of multiple triplets of spheres that form complex hierarchical structures. The double oscillating-chain phases are formed by packing triplets with alternating chirality, resulting in the cancellation of the global chirality. It is merely a slight change in the anisotropy of the cylinder that brings about these changes. In circular cylinders, the densest structure transitions from zigzag to helical phases as the diameter increases, whereas in elliptic cylinders, the transition involves intermediate double oscillated-chain phases. This can be understood simply: when the minor axis is sufficiently large to form a helix, increasing the aspect ratio provides more space along the major axis, causing the spheres to separate toward both ends. However, because the available space at the ends is limited, a complete helical structure cannot be formed, forcing the spheres into oscillatory arrangements.

This work reveals the surprising complexity that emerges from an impressively simple system. Using elliptic tubes as the typical geometry, we show that even infinitesimal anisotropy can lead to entirely new phases—an effect we anticipate will be ubiquitous across a broad spectrum of anisotropic confinements. The predictions have been realized by our macroscopic experiments and are expected to encourage further experiments across many length scales. The findings will inevitably stimulate future explorations of richer interactions from atoms and nanoparticles to biological morphogenesis and even macroscopic phenomena in anisotropic confinements, and promise to yield more intriguing packing structures. A compelling illustration is our ongoing project on water encapsulated in elliptical carbon nanotubes, which already exhibits several of the packing structures presented here. Our work gives a new way to design materials through packings under confinement, and helps researchers to understand many puzzles in living systems.

## Methods

### Model

We set the sphere diameter to *d* = 1 (unit length), with all other quantities expressed relative to *d*. For an elliptic cylinder, the major axis is denoted by *D*_*a*_ and the minor axis by *D*_*b*_; the aspect ratio *D*_*a*_/*D*_*b*_ quantifies the elliptical cross-sectional flattening. The cross-sectional plane lies in the *xy*-plane, and the cylinder’s axis is aligned along the *z*-axis. Circular cylinders necessitate periodic boundary conditions along the *z*-axis and twisted periodic boundary conditions in the *xy*-plane, owing to their invariance under arbitrary rotations about the central axis. In contrast, elliptic cylinders are anisotropic in the *xy*-plane; thus, only periodic boundary conditions along the *z*-axis are used, without twisted periodic conditions. However, this inevitably increases the influence of both the periodic boundary conditions and the finite number of spheres on the search for the densest packings. We focus on slightly elliptic cylinders with minor axis ${D}_{b}\in \left[1.6,2\right]$ and aspect ratios ${D}_{a}/{D}_{b}\in \left[1,1.10\right]$, and employ the Monte Carlo (MC) method with simulated annealing for sphere numbers $N\in \left[13,25\right]$. All cases with *N *≤ 12 are well covered, since they are the factors of $N\in \left[13,25\right]$.

### Simulation process

We employ the Monte Carlo method with simulated annealing, where particles move according to the Metropolis algorithm. The overlap energies between two spheres, ${E}_{ij}^{{{{{\rm{S}}}}}}$, and between a sphere and the wall, ${E}_{i}^{{{{{\rm{B}}}}}}$, are defined as

1 $${E}_{ij}^{{{{{\rm{S}}}}}}=\left\{\begin{array}{ll}\frac{1}{2}{({r}_{ij}-d)}^{2}\quad &{r}_{ij}\le d\\ 0\quad\hfill &{r}_{ij} > d\end{array}\right.,$$

and

2 $${E}_{i}^{{{{{\rm{B}}}}}}=\left\{\begin{array}{ll}\frac{1}{2}{\left({r}_{i{{{{\rm{B}}}}}}-\frac{d}{2}\right)}^{2}\quad &{r}_{i{{{{\rm{B}}}}}}\le \frac{d}{2}\\ 0\hfill\quad &{r}_{i{{{{\rm{B}}}}}} > \frac{d}{2}\end{array}\right..$$

Here, *r*_*ij*_ is the distance between the centers of two different hard spheres, and *r*_*i*B_ is the distance between a sphere’s center and the elliptic cylinder wall. It is worth noting that when the total energy is zero, there is no overlap between the spheres and between the spheres and the wall, and only then do the spheres become truly hard spheres. By adjusting the cylinder height, we are effectively searching for the case in which the spheres just separate from each other and from the wall. At this point, the spheres become hard spheres, and the resulting structure corresponds to a possible densest packing structure^8^.

We consider a series of elliptic cylinders with various minor axes *D*_*b*_ and aspect ratios *D*_*a*_/*D*_*b*_ to obtain the densest packing structure for each case. For a specific *D*_*b*_ and *D*_*a*_/*D*_*b*_, the simulation process is as follows:

1. For a given number of hard spheres *N* and a cylinder length *L*, simulated annealing is used to obtain the optimal solution (the minimum total energy).
2. The length *L* is then varied to find the minimum *L* at which the total energy is zero (spheres and wall are just separated), and the packing fraction *ϕ* is computed.
3. The procedure is repeated for different *N* to find the sphere number corresponding to the maximum *ϕ*.
4. Multiple runs (at least five per case) are performed, and the optimal result is taken as the possible densest packing for the corresponding *D*_*b*_ and *D*_*a*_/*D*_*b*_.

In this work, the minor axis *D*_*b*_ of the elliptic cylinder is in the range ${D}_{b}\in \left[1.6,2\right]$. This range corresponds to the zigzag, single-helix, and double-helix phases observed in the circular cylinder (viewed as an elliptic cylinder with *D*_*a*_/*D*_*b*_ = 1, where *D*_*b*_ is the circular cylinder diameter *D*). An equal number of experimental points are taken from each of the three phases to avoid the issue of uniform sampling leading to too few points in certain phases. For aspect ratios ${D}_{a}/{D}_{b}\in \left[1,1.10\right]$, since the focus is on elliptic cylinders approaching the circular cylindrical limit, more points are sampled when *D*_*a*_/*D*_*b*_ is small and fewer when it is large; practically, uniformly selecting points based on the eccentricity $e=\sqrt{1-{\left(\frac{{D}_{b}}{{D}_{a}}\right)}^{2}}$ fulfills this requirement. Further details are provided in Section H of the Supplemental Material See supplemental material.

### The triplet-sphere packing block method

Based on the theory of packing in circular cylinders^15^, the helix phases feature spheres with a coordination number of 4, where every three spheres contact both the wall and one another, forming triplets of spheres [Fig. 2a]. In the helical phases of elliptic cylinders, triplets of spheres still exist. A polar coordinate system in the *xy*-plane, with the ellipse’s center as the origin, is used to specify sphere positions via the angular coordinate θ and the radial distance $r\left(\theta \right)$. Consider three spheres positioned at ${r}_{i}\triangleq r\left({\theta }_{i}\right)$, ${r}_{i+1}\triangleq r\left({\theta }_{i+1}\right)$, and ${r}_{i+2}\triangleq r\left({\theta }_{i+2}\right)$, with angular differences Δθ_*i*,*i*+1_, Δθ_*i*+1,*i*+2_, and Δθ_*i*,*i*+2_. When these spheres form a triplet of spheres with heights *z*_*i*_ < *z*_*i*+1_ < *z*_*i*+2_ (from the top view, a counterclockwise arrangement of spheres *i*, *i* + 1, *i* + 2 corresponds to a right-handed triplet; otherwise, it is left-handed), the following condition is satisfied (see derivation in Section B of the Supplemental Material See supplemental material):

3 $$\begin{array}{c}\sqrt{{d}^{2}-{\left({r}_{i}-{r}_{i+2}\right)}^{2}-4{r}_{i}{r}_{i+2}{\sin }^{2}\frac{\Delta {\theta }_{i,i+2}}{2}}\\=\sqrt{{d}^{2}-{\left({r}_{i}-{r}_{i+1}\right)}^{2}-4{r}_{i}{r}_{i+1}{\sin }^{2}\frac{\Delta {\theta }_{i,i+1}}{2}}\\+\sqrt{{d}^{2}-{\left({r}_{i+1}-{r}_{i+2}\right)}^{2}-4{r}_{i+1}{r}_{i+2}{\sin }^{2}\frac{\Delta {\theta }_{i+1,i+2}}{2}}\end{array}.$$

Given θ_*i*_ and Δθ_*i*,*i*+1_, solving the equation determines Δθ_*i*+1,*i*+2_, yielding θ_*i*+1_ ≜ θ_*i*_ + Δθ_*i*,*i*+1_ and θ_*i*+2_ ≜ θ_*i*_ + Δθ_*i*,*i*+1_ + Δθ_*i*+1,*i*+2_. The vertical height differences Δ*z*_*i*,*i*+1_, Δ*z*_*i*+1,*i*+2_ and Δ*z*_*i*,*i*+2_ can then be calculated. By Eq. (3), the positions of the *i*-th and $\left(i+1\right)$-th spheres entirely determine the position of the $\left(i+2\right)$-th sphere, and subsequently determine the packing of *N* spheres. So we can vary the parameters θ_1_ and Δθ_1,2_ to generate distinct configurations and then seek out the one that yields the maximum packing fraction. We call this approach the triplet-sphere packing block method.

## Supplementary information

Supplementary Information

Transparent Peer Review file

## Data Availability

The source data for Figs. 1, 3–5 and other raw data are deposited in Zenodo under accession code 10.5281/zenodo.20590037.

## Code availability

The simulation codes used in this study are deposited in Zenodo under accession code 10.5281/zenodo.20590037.
