# Supplementary material for: Chiral packings in cylinders are ultrasensitive to confinement deformation
Source: Nat Commun. 2026 Jul 16;17:6398. doi: 10.1038/s41467-026-74709-2 (PMC13376757; doi:10.1038/s41467-026-74709-2)
Supplement: Supplementary file 1 — Supplementary Information [file 41467_2026_74709_MOESM1_ESM.pdf]

Supplemental Material: Chiral Packings in Cylinders are Ultrasensitive to Confinement Deformation

Xuebin Wang,[1] Jiahao Guo,[1] and Yao Li[1,*]

[1]*School of Physics and Key Laboratory of Functional Polymer Materials of Ministry of Education, Nankai University, and Collaborative Innovation Center of Chemical Science and Engineering, Tianjin 300071, China*

(Dated: June 8, 2026)

A. Zigzag Structure

For clarity in subsequent discussions, we define the semi-major axis of the ellipse as $a \triangleq D_a/2$ and the semi-minor axis as $b \triangleq D_b/2$. Within an elliptic cylinder, the major axis provides more space; therefore, the zigzag structure is necessarily arranged along the major axis. The distance between two adjacent spheres in the xy-plane is $2a - d$, and the vertical difference is denoted by Δz, which satisfies

$$\Delta z^2 + (2a - d)^2 = d^2. \tag{S1}$$

Thus,

$$\Delta z = \sqrt{4ad - 4a^2}. \tag{S2}$$

Accordingly, the packing fraction is given by

$$\phi = \frac{\frac{4}{3}\pi \left(\frac{d}{2}\right)^3}{\pi ab\sqrt{4ad - 4a^2}}. \tag{S3}$$

When the minor axis D_b remains constant while the aspect ratio D_a/D_b increases, or when D_a/D_b is fixed and the minor axis D_b increases, the major axis will become larger. When the major axis length reaches $\left(1 + \frac{\sqrt{3}}{2}\right) d$, the number of neighbors per sphere changes from 2 to 4, giving

$$\frac{D_a}{D_b} = \frac{a}{b} = \frac{\frac{\left(1+\frac{\sqrt{3}}{2}\right)d}{2}}{\frac{D_b}{2}}. \tag{S4}$$

Thus, the boundary of the zigzag structure is given by

$$D_b = \frac{2 + \sqrt{3}}{2} d \times \frac{D_b}{D_a}. \tag{S5}$$

B. Analytical Expressions for Triplets of Spheres

We first derive the contact condition between two spheres. In the triplet configuration, each sphere maintains contact with the elliptic cylinder wall. A polar coordinate system is established in the xy-plane with the center of the ellipse as the origin. The angular position of a hard sphere's center is denoted by θ, and its distance from the origin is $r(\theta)$. As θ varies from 0 to 2π, $r(\theta)$ traces an approximately elliptical path. Consider two spheres in the xy-plane

* liyao@nankai.edu.cn

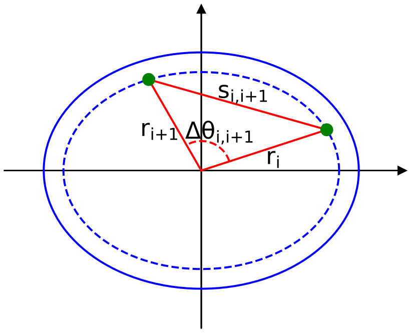

Figure S1. Top view of two spheres in contact within an elliptic cylinder. The blue solid line represents the elliptic cylinder wall, and the blue dashed line indicates the trajectory of the hard spheres' centers. The green points mark the centers of the two contacting spheres, which, together with the origin, form a triangular structure.

located at $r_i \triangleq r(\theta_i)$ and $r_{i+1} \triangleq r(\theta_{i+1})$, respectively, with an angular difference $\Delta\theta_{i,i+1}$ (see Fig. S1). Their distance in the xy-plane is given by

$$s_{i,i+1} \triangleq \sqrt{r_i^2 + r_{i+1}^2 - 2r_i r_{i+1} \cos \Delta\theta_{i,i+1}}. \tag{S6}$$

Let $\Delta z_{i,i+1}$ be the vertical separation along the z-axis. When the two spheres are in contact, they satisfy

$$(\Delta z_{i,i+1})^2 + (s_{i,i+1})^2 = d^2. \tag{S7}$$

Thus, the contact condition between the two spheres becomes

$$(\Delta z_{i,i+1})^2 + (r_i - r_{i+1})^2 + 4r_i r_{i+1} \sin^2 \frac{\Delta\theta_{i,i+1}}{2} = d^2. \tag{S8}$$

In the case of a circular cylindrical confinement, the isotropy in the xy-plane ensures that the radial distance is independent of θ; hence, $r_i = r_{i+1} = \frac{D-1}{2}$ (with D as the cylinder diameter). Consequently, from the previous theory [1], Eq. S8 reduces to

$$(\Delta z_{i,i+1})^2 + (D-1)^2 \sin^2 \frac{\Delta\theta_{i,i+1}}{2} = d^2. \tag{S9}$$

When three spheres are mutually contacting, and if their z coordinates satisfy $z_i < z_{i+1} < z_{i+2}$, then

$$\Delta z_{i,i+2} = \Delta z_{i,i+1} + \Delta z_{i+1,i+2}, \tag{S10}$$

$$\Delta\theta_{i,i+2} = \Delta\theta_{i,i+1} + \Delta\theta_{i+1,i+2}. \tag{S11}$$

Substituting the two-sphere contact condition Eq. S8 into Eq. S10, we obtain (i.e. Eq. 3 in the main text)

$$\begin{aligned} &\sqrt{d^2 - (r_i - r_{i+2})^2 - 4r_i r_{i+2} \sin^2 \tfrac{\Delta\theta_{i,i+2}}{2}} \\ = &\sqrt{d^2 - (r_i - r_{i+1})^2 - 4r_i r_{i+1} \sin^2 \tfrac{\Delta\theta_{i,i+1}}{2}} \\ + &\sqrt{d^2 - (r_{i+1} - r_{i+2})^2 - 4r_{i+1} r_{i+2} \sin^2 \tfrac{\Delta\theta_{i+1,i+2}}{2}} \end{aligned}. \tag{S12}$$

Similarly, in the circular cylindrical case [1], Eq. S12 becomes

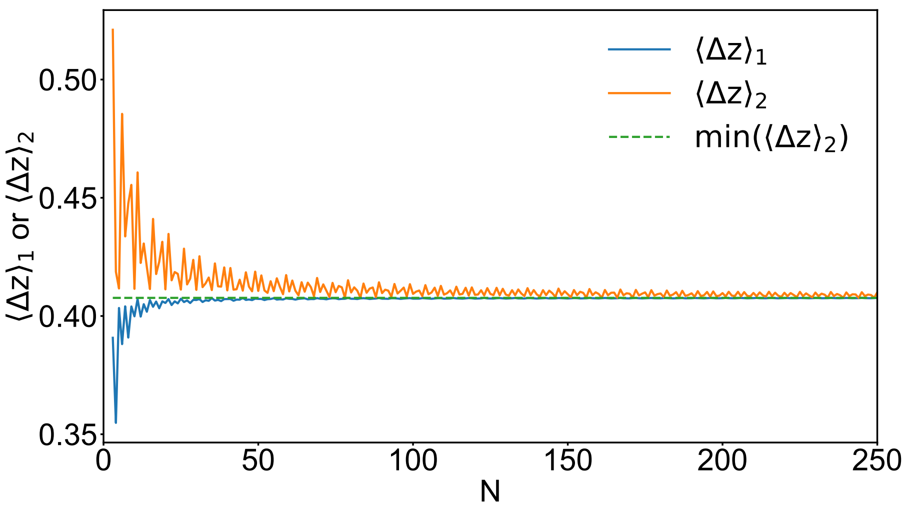

Figure S2. For structures with different numbers of spheres N ($N \leq 250$), we compare $\langle \Delta z \rangle_1$, $\langle \Delta z \rangle_2$, and the minimum $\langle \Delta z \rangle_2$. For large N, the minimum $\langle \Delta z \rangle_2$ and $\langle \Delta z \rangle_1$ are nearly identical, indicating the densest packing structure.

$$\sqrt{d^2 - (D-1)^2 \sin^2 \frac{\Delta\theta_{i,i+2}}{2}} = \sqrt{d^2 - (D-1)^2 \sin^2 \frac{\Delta\theta_{i,i+1}}{2}} + \sqrt{d^2 - (D-1)^2 \sin^2 \frac{\Delta\theta_{i+1,i+2}}{2}}. \tag{S13}$$

By varying $\Delta\theta_{i,i+1}$, the local minimum of $\Delta z_{i,i+2}$ can be identified. In the single helix configuration, $\Delta z_{i,i+2}$ exhibits a single local minimum, whereas in the double helix configuration it exhibits two. These minima correspond to one type of triplet of spheres in the single helix and two types in the double helix.

C. Supplementary explanation of the Triplet-sphere Packing Block Method

According to Eq. S12 (i.e. Eq. 3 in the main text), the positions of the i-th and $(i+1)$-th spheres determine the position of the $(i+2)$-th sphere. Therefore, given θ_1 and $\Delta\theta_{1,2}$, one can construct a certain packing of N spheres. The average vertical height difference between adjacent spheres is $\langle \Delta z \rangle \triangleq \frac{\sum_{i=1}^{N-1} \Delta z_{i,i+1}}{N-1}$. By varying θ_1 and $\Delta\theta_{1,2}$, one obtains various packings and different $\langle \Delta z \rangle$. To find the minimum $\langle \Delta z \rangle$, we first sweep θ_1 and $\Delta\theta_{1,2}$ over a broad range, identify the point yielding the smallest $\langle \Delta z \rangle$, and then center the point within a smaller domain to search for the smallest $\langle \Delta z \rangle$ again. We repeat this process until the region's side length falls below 10^{-5}, and record the center point that minimizes $\langle \Delta z \rangle$.

For structures composed of N spheres, we define $\langle \Delta z \rangle_1 \triangleq \min\left(\frac{\sum_{i=1}^{N-1} \Delta z_{i,i+1}}{N-1}\right)$, which represents the minimum average vertical height difference between adjacent spheres. As $N \to \infty$, $\langle \Delta z \rangle_1$ corresponds to the densest packing configuration. However, in computations, sphere numbers cannot be infinite, and $\langle \Delta z \rangle_1$ fails to describe simulation results under finite N. Since periodic boundary conditions along the z-axis are used in the simulation, their effect must be considered by using $\langle \Delta z \rangle_2 \triangleq \min\left(\frac{\sum_{i=1}^{N-1} \Delta z_{i,i+1} + \Delta z_{1,N}}{N}\right)$, where $\Delta z_{1,N}$ represents the contribution from the periodic boundary. For $N \leq N_{max}$ (where N_{max} is any integer), each N yields a distinct $\langle \Delta z \rangle_2$. We identify the minimum $\langle \Delta z \rangle_2$, which represents the periodic unit of the possible densest packing under $N \leq N_{max}$. When N_{max} is sufficiently large, this minimum $\langle \Delta z \rangle_2$ corresponds to the true densest packing. As shown in Fig. S2, $\langle \Delta z \rangle_1$, $\langle \Delta z \rangle_2$, and the minimum $\langle \Delta z \rangle_2$ across all N ($N \leq 250$) are plotted. At small N, $\langle \Delta z \rangle_2$ significantly exceeds $\langle \Delta z \rangle_1$, but for large N, the minimum $\langle \Delta z \rangle_2$ is almost the same as $\langle \Delta z \rangle_1$. This demonstrates that the minimum $\langle \Delta z \rangle_2$ reliably represents the densest packing configuration for large N, while its accuracy diminishes for small N.

The optimal number of spheres N^* is the value of N (for $N \leq N_{max}$) that yields the minimum $\langle \Delta z \rangle_2$.Table S1 compares N^* at $D_b = 1.940256$ (helical phase I) for various aspect ratios D_a/D_b: simulation results ($N \leq 25$) versus theoretical predictions ($N \leq 25$ and $N \leq 6400$). With periodic boundary conditions, the theoretical predictions for $N^* = 5$ correspond to simulation results for $N^* = 20$ and $N^* = 25$. For $N \leq 25$, most theoretical predictions agree with simulation results, demonstrating that the triplet-sphere packing block method indeed identifies the densest

D_a/D_b	Simulation ($N \leq 25$)	Theory ($N \leq 25$)	Theory ($N \leq 6400$)
1.00020	22	22	3597
1.00080	22	22	5823
1.00180	22	22	4266
1.00322	22	22	4139
1.00504	22	22	3197
1.00728	22	22	1238
1.00995	24	24	3011
1.01305	20	5	3233
1.01660	25	5	5006
1.02062	25	25	1635
1.02512	25	25	4397
1.03011	21	25	4042
1.03562	23	23	5928
1.04167	23	23	3758

Table S1. In helical phase I at $D_b = 1.940256$, a comparison of the optimal number of spheres N^* between theoretical predictions and simulation results is presented.

helical structures reliably. For theoretical predictions with $N \leq 6400$, the optimal number of spheres N^* becomes larger than in the case of $N \leq 25$, suggesting that a genuine period of the densest structure may have been identified.

We can give a simple validation of the triplet-sphere packing block method. Although this method is proposed to explain the packing structures in elliptic cylinders, a circular cylinder can also be regarded as a special case of an elliptic cylinder with $D_a = D_b$. Therefore, the triplet-sphere packing block method is still valid. In the Fig. S3, we plot the packing fraction as a function of the cylinder diameter using the analytical expressions for the helical phases in the circular cylinder reported in previous studies [1] (solid line) as well as the results obtained by the triplet-sphere packing block method with $N \leq 6400$ (green dashed line). The two curves almost coincide, indicating that the triplet-sphere packing block method can obtain the densest helical structures in the circular cylinder. In our simulation, since twisted periodic boundary conditions are not used, the obtained structures do not reach the densest structures, and their packing fractions (the points in Fig. S3) are significantly lower than those of the densest structures. However, the results from the triplet-sphere packing block method with $N \leq 25$ (blue dashed line) still agree well with the simulation results. It is worth noting that the last four simulation points actually form the helical phase II because twisted periodic boundary conditions are not used. The corresponding structure should be obtained by the defective triplet-sphere packing block method, but here we also include it in the triplet-sphere packing block method. In conclusion, the triplet-sphere packing block method is still valid for the cylinder and has been preliminarily verified.

D. Further analysis of the effect of periodic boundary conditions on helical structures

To further clarify the influence of the periodic boundary conditions on the hierarchical periodic structure of the helical phase, we perform a detailed analysis of Fig. 5 in the main text and discuss the four cases A, B, C and D below.

It can be seen from Fig. 3(a) in the main text that, in the general case, the simulation results are substantially different from the theoretical densest packings. Taking case A as an example, at $D_b = 1.940256$ and $D_a/D_b = 1.02512$ [dashed A in Fig. 3(a)], we use the triplet-sphere packing block method for sphere numbers $N \leq 25$, reproduce the simulation results, and determine the optimal sphere number $N^* = 25$. The optimal sphere number represents the period of the constructed structure, so by using periodic boundary conditions, we can obtain the angular positions of the first 50 spheres, as shown in Fig. 5(a). It can be seen that every five spheres still form a subperiod, and the angular offset of each sphere persists. However, after one offset, the angular positions of the five spheres in the last subperiod of one period differ from those of the five spheres in the first subperiod, indicating that the sphere positions undergo a discontinuous change at the period boundary. In Fig. 5(a), the left red arrow indicates the angular change of one sphere across subperiods within an period; the right red arrow denotes the left red arrow in the next period; the blue arrows represent the angular changes of the two spheres whose angles are closest to that sphere in the subperiod. The spheres marked by the left red arrow are not collinear with those marked by the three right arrows, illustrating the

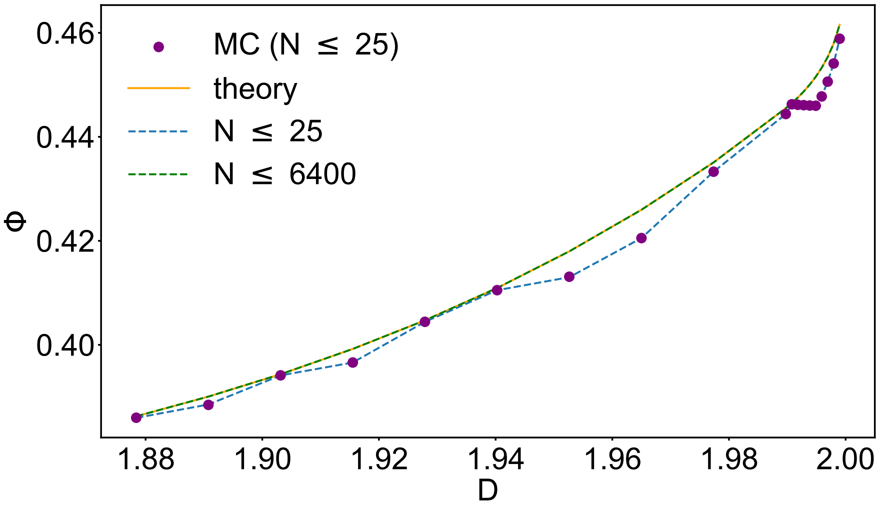

Figure S3. The points represent the MC simulation results for $N \leq 25$. The solid line represents the analytical expressions for the helical phases in the circular cylinder, as reported in previous studies, while the dashed lines show the results obtained by the triplet-sphere packing block method with $N \leq 25$ (blue dashed line) and $N \leq 6400$ (green dashed line).

sudden change in angular positions caused by the period boundary. Compared with the theoretical helical structure for the same parameters [Fig. 4(a)], finite-size periodic boundary conditions have a strong effect at small sphere numbers and disrupt the hierarchical periodic structures. Nevertheless, it is possible to obtain structures close to the densest packings for small sphere numbers, as shown by dashed lines B, C, and D in Fig. 3(a).

For case B, the aspect ratio is $D_a/D_b = 1.018554$, and we find the optimal sphere number $N^* = 5$. Similarly, applying periodic boundary conditions gives the angular positions of the first 50 spheres shown in Fig. 5(b). Since the optimal number of spheres is $N^* = 5$, the subperiod composed of five spheres becomes the period of the structure. This indicates that the angular positions of the spheres do not shift after one subperiod, which is demonstrated by the two red arrows being collinear in the figure. At $D_b = 1.940256$, for the densest packings (constructed by the triplet-sphere packing block method when $N \leq 6400$), we investigate the average-angular offset of the spheres between subperiods, $\overline{\Delta\theta_{k,k+5}}$, under various aspect ratios, as shown in Fig. 5(e). When $D_a/D_b \approx 1.019$ (red dashed line), the average-angular offset is zero, implying that the period of the theoretical densest helical structure is five at this aspect ratio. Since case B is near the red dashed line in Fig. 5(e), the average-angular offset of the theoretical densest packing is approximately zero. Thus, even if the sphere number in the constructed structure is large, the total angular offset of the spheres is tiny, making the overall structure relatively close to a structure with period five. Therefore, for $N \leq 25$, the constructed structure has a period of five and a packing fraction close to the densest packing.

For case C, the aspect ratio is $D_a/D_b = 1.000450$, and the optimal sphere number is $N^* = 22$. Similarly, the angular positions of the first 50 spheres are shown in Fig. 5(c). Here, the spheres marked by the left red arrow are collinear with those marked by the upper right blue arrow. This indicates that, by offsetting one subperiod, the angular positions of the last five spheres of a period coincide with those of the five spheres in the first subperiod of that period. Consequently, two distinct sequences formed by the spheres with two different serial numbers within each subperiod in consecutive periods are collinear. Specifically, the sequences formed by the 1st, 2nd, 3rd, 4th, and 5th spheres within each subperiod of one period are respectively collinear with the sequences formed by the 4th, 5th, 1st, 2nd, and 3rd spheres within each subperiod of the subsequent period. The collinear alignment reduces the influence of periodic boundary conditions, thereby enabling a hierarchical periodic structure at small sphere numbers. Therefore, for $N \leq 25$, the packing fraction of the constructed structure is close to the theoretical densest value. For case D, the aspect ratio is $D_a/D_b = 1.041667$, and the optimal sphere number is $N^* = 23$. The angular positions of the first 50 spheres are shown in Fig. 5(d). Unlike case C, the spheres marked by the left red arrow are collinear with those marked by the lower right blue arrow. The sequences formed by the 1st, 2nd, 3rd, 4th, and 5th spheres within each subperiod of one period are respectively collinear with the sequences formed by the 3rd, 4th, 5th, 1st, and 2nd spheres within each subperiod of the subsequent period. Similarly, the collinear alignment perfectly connects the structures of adjacent periods and yields a packing close to the densest structure for small sphere numbers.

To better understand cases C and D, we consider the total angular offset of a certain serial number sphere of the subperiod over one period. If this total offset equals the angular difference between two spheres within the subperiod, the corresponding spheres of the structures in consecutive periods are collinear. Fig. 5(f) presents the angular difference $\Delta\theta_{2,22}$ between the 22nd and 2nd spheres in the structure at various aspect ratios when the sphere

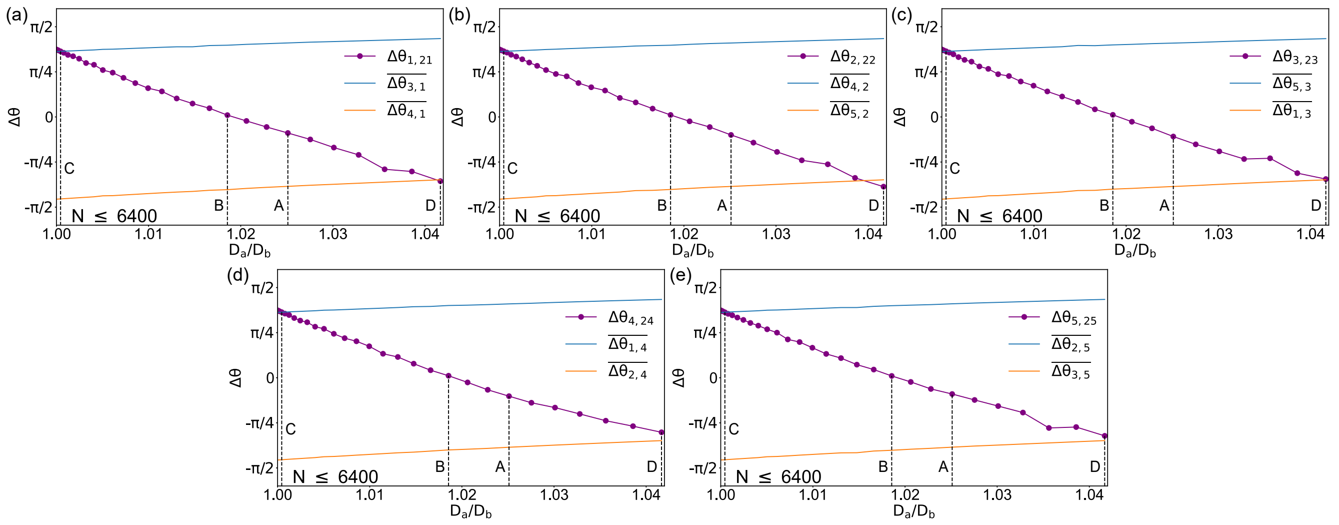

Figure S4. For theoretical packing structures (constructed for $N \leq 6400$): (a) We plot the angular difference $\Delta\theta_{1,21}$ between the 1st and 21st spheres as a function of aspect ratio, together with the average-angular differences between the 1st and 3rd spheres, $\overline{\Delta\theta_{3,1}}$, and between the 1st and 4th spheres, $\overline{\Delta\theta_{4,1}}$, within the subperiods. (b) We plot $\Delta\theta_{2,22}$ and compare it with $\overline{\Delta\theta_{4,2}}$ and $\overline{\Delta\theta_{5,2}}$, which correspond to the average-angular differences between the 2nd and 4th spheres, and between the 2nd and 5th spheres, respectively, within the subperiods. (c)–(e) Analogous plots are shown for $\Delta\theta_{3,23}$, $\Delta\theta_{4,24}$, and $\Delta\theta_{5,25}$, together with the corresponding average-angular differences of spheres within the subperiods.

number $N \leq 25$, and compares it with the average-angular differences between the 2nd and 4th spheres, $\overline{\Delta\theta_{4,2}}$, as well as between the 2nd and 5th spheres, $\overline{\Delta\theta_{5,2}}$, within the subperiods. In Fig. S4(b), we also plot the angular-difference behavior of the theoretical packing structures (constructed for $N \leq 6400$). Compared with Fig. 5(f), the curves follow the same trends in most regions. This indicates that the structure constructed with a small number of spheres already conforms to the theoretical structure, indirectly confirming the significant influence of periodic boundary conditions on the overall structure. For the structure constructed for $N \leq 25$, when $\Delta\theta_{2,22}$ approaches $\overline{\Delta\theta_{4,2}}$, the total angular offset of the 2nd sphere within the subperiods over one period almost equals the average-angular difference between the 2nd and 4th spheres within the subperiods. This indicates that in the theoretically densest structure, the angular difference between the 2nd and 22nd spheres equals that between the 22nd and 24th spheres (the 21st to 25th spheres form one subperiod), causing the 24th sphere to occupy the same angular position as the 2nd sphere. Thus, the period of the overall structure is 22. Similarly, when $\Delta\theta_{2,22}$ approaches $\overline{\Delta\theta_{5,2}}$, the 25th sphere and the 2nd sphere share the same angular position in the densest structure, which corresponds to the overall structure having a period of 23. Fig. 5(f) shows that case C lies in the region where $\Delta\theta_{2,22}$ approaches $\overline{\Delta\theta_{4,2}}$, while case D lies in the region where $\Delta\theta_{2,22}$ approaches $\overline{\Delta\theta_{5,2}}$. In addition, Fig. S4 shows the comparison of various angular differences in the theoretical densest packings with the corresponding average-angular differences of spheres within the subperiods. Due to the anisotropy of the cylinders, the crossing positions of the curves differ slightly among these figures, but case C is located in the region where the curves approach each other on the left, and case D is located in the region where the curves approach each other on the right. Hence, the optimal sphere numbers of the structures constructed by cases C and D for $N \leq 25$ are 22 and 23, respectively, with packing fractions very close to the theoretical densest values.

E. Another method to solve the Double Oscillated-Chain

In the double oscillated-chain ($s = 2$), every four spheres form a periodic unit, and the structure can be regarded as a combination of two zigzag arrangements. From the top view, the structure is symmetric with respect to both the x- and y-axes. As shown in Fig. S5(a), the center of a sphere in the first quadrant is located at (p, q). Assume that this sphere contacts the wall of the elliptic cylinder at the point (x, y), where

$$\left(\frac{x}{a}\right)^2 + \left(\frac{y}{b}\right)^2 = 1. \tag{S14}$$

Define the squared distance between the sphere's center and the wall as $s^2 \triangleq (x-p)^2 + (y-q)^2$. Because the

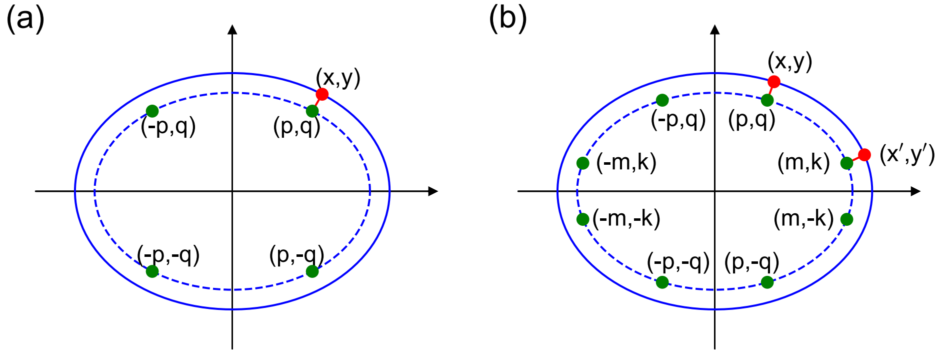

Figure S5. Top-view sketches of the double oscillated-chain structures for $s = 2$ and $s = 3$ are provided. The blue solid line represents the elliptic cylinder wall, the blue dashed line indicates the trajectory of the hard spheres' centers, the green dots denote the positions of the hard spheres' centers, and the red dots mark the contact points between the spheres and the wall. (a) For the double oscillated-chain ($s = 2$), one period consists of four spheres. Assuming that a sphere in the first quadrant is located at $(p,\, q)$ and contacts the elliptic cylinder wall at $(x,\, y)$, the other sphere positions are obtained by symmetry. (b) For the double oscillated-chain ($s = 3$), one period consists of eight spheres. Assuming that two spheres in the first quadrant are located at $(p,\, q)$ and $(m,\, k)$, and contact the elliptic cylinder wall at $(x,\, y)$ and $(x',\, y')$, respectively, the remaining sphere positions can also be obtained by symmetry.

sphere is exactly in contact with the wall, the minimum value of s^2 must be $\left(\frac{d}{2}\right)^2$. That is,

$$\begin{aligned} s^2 &= (x-p)^2 + (y-q)^2 = (x-p)^2 + \left(b\sqrt{1-\left(\tfrac{x}{a}\right)^2} - q\right)^2 \\ &= \left(1-\tfrac{b^2}{a^2}\right)x^2 - 2px - 2bq\sqrt{1-\tfrac{x^2}{a^2}} + p^2 + b^2 + q^2 = \left(\tfrac{d}{2}\right)^2 \end{aligned}, \tag{S15}$$

$$\frac{d\left(s^2\right)}{dx} = 2\left(1-\frac{b^2}{a^2}\right)x - 2p + \frac{2bqx}{a^2\sqrt{1-\frac{x^2}{a^2}}} = 0. \tag{S16}$$

From the triplet of spheres, we know that $\Delta z_{1,3} = \Delta z_{1,2} + \Delta z_{2,3}$, with $\Delta z_{1,2} = \sqrt{d^2 - 4\left(p^2+q^2\right)}$, $\Delta z_{2,3} = \sqrt{d^2 - 4p^2}$ and $\Delta z_{1,3} = \sqrt{d^2 - 4q^2}$, Thus,

$$\sqrt{d^2 - 4q^2} = \sqrt{d^2 - 4\left(p^2+q^2\right)} + \sqrt{d^2 - 4p^2}. \tag{S17}$$

By simultaneously solving Eq. S15, Eq. S16, and Eq. S17, the coordinates $(p,\, q)$ are directly obtained, meaning that the double oscillated-chain ($s = 2$) structure is directly solvable.

Similarly, in the double oscillated-chain ($s = 3$), every eight spheres form a periodic unit, and the structure is also symmetric with respect to both the x- and y-axes. As shown in Fig. S5(b), two spheres in the first quadrant are located at $(p,\, q)$ and $(m,\, k)$, respectively. Assume they contact the elliptictails in Section B cylinder wall at $(x,\, y)$ and $(x',\, y')$. The sphere at $(p,\, q)$ still satisfies Eq. S15 and Eq. S16, and by replacing p, q, and x with m, k, and x' , the sphere at $(m,\, k)$ also satisfies these equations. From the triplet of spheres, we have $\Delta z_{1,3} = \Delta z_{1,2} + \Delta z_{2,3}$ and $\Delta z_{2,4} = \Delta z_{2,3} + \Delta z_{3,4}$, with $\Delta z_{1,2} = \sqrt{d^2 - 4\left(p^2+q^2\right)}$, $\Delta z_{2,3} = \sqrt{d^2 - \left[(p+m)^2 + (q+k)^2\right]}$, $\Delta z_{1,3} = \sqrt{d^2 - \left[(p-m)^2 + (q-k)^2\right]}$, $\Delta z_{3,4} = \sqrt{d^2 - 4m^2}$ and $\Delta z_{2,4} = \sqrt{d^2 - \left[(p-m)^2 + (q+k)^2\right]}$. Thus, we obtain

$$\sqrt{d^2 - \left[(p-m)^2 + (q-k)^2\right]} = \sqrt{d^2 - 4\left(p^2+q^2\right)} + \sqrt{d^2 - \left[(p+m)^2 + (q+k)^2\right]}, \tag{S18}$$

$$\sqrt{d^2 - \left[(p-m)^2 + (q+k)^2\right]} = \sqrt{d^2 - \left[(p+m)^2 + (q+k)^2\right]} + \sqrt{d^2 - 4m^2}. \tag{S19}$$

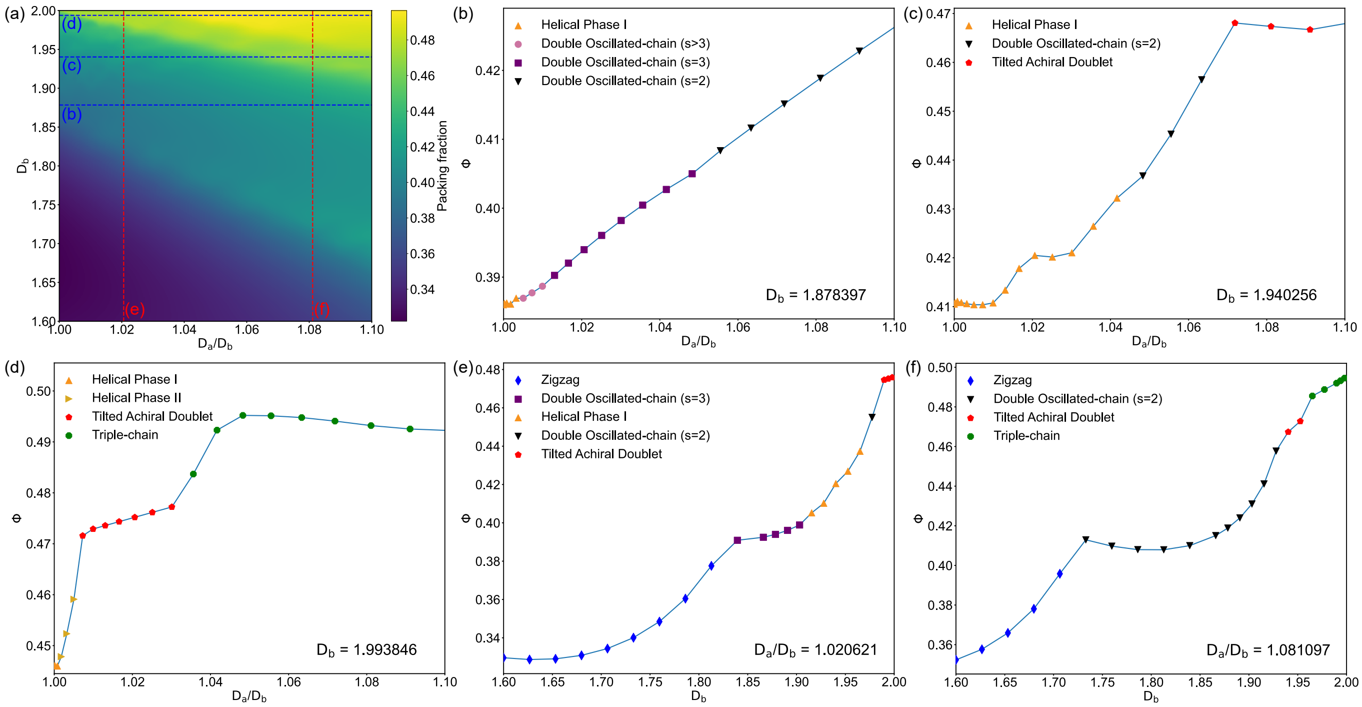

Figure S6. The packing fraction of simulations at each point in the phase diagram is color-coded to produce the 2D color map in panel (a). With the minor axis D_b fixed, the variation of the packing fraction ϕ with aspect ratio D_a/D_b is shown in panels (b), (c), and (d). With the aspect ratio D_a/D_b fixed, the variation of the packing fraction ϕ with minor axis D_b is shown in panels (e) and (f).

By solving Eqs. S15, S16, S18, and S19, both $(p,\, q)$ and $(m,\, k)$ can be determined, which give the double oscillated-chain $(s = 3)$ structure.

Unlike the approach of constructing the double oscillated-chain using sphere packing of triplets of spheres, this method exploits the symmetry with respect to the x-axis and y-axis to directly solve the triplets of spheres and thereby determine the double oscillated-chain structure. Therefore, the packing fractions of the double oscillated-chain $(s = 2)$ and double oscillated-chain $(s = 3)$ can be computed rapidly and accurately, thereby yielding the boundary line between the two structures.

F. Packing Fractions

Fig. S6(a) presents a two-dimensional color map of the packing fraction ϕ for simulations with sphere number $N \leq 25$. Color is used to indicate variations in the packing fraction ϕ. In Fig. S6(a), the packing fraction ϕ is clearly low in the lower-left region and high in the upper-right, indicating that increasing both the minor axis D_b and the aspect ratio D_a/D_b significantly raises ϕ. To investigate the variation trends in the packing fraction, we keep the minor axis D_b fixed and plot the packing fraction ϕ as a function of the aspect ratio D_a/D_b in Figs. S6(b), S6(c), and S6(d), where ϕ generally increases but sometimes decreases slightly. Similarly, we keep the aspect ratio D_a/D_b fixed and plot the packing fraction ϕ as a function of the minor axis D_b in Figs. S6(e) and S6(f), where ϕ also exhibits an overall rise with occasional small decreases. In these two cases, we find that when increasing either the aspect ratio D_a/D_b or the minor axis D_b, ϕ increases substantially upon a phase change but may decrease slightly when no phase change occurs. As either the minor axis D_b or the aspect ratio D_a/D_b increases, the cylinder's volume per unit length expands. When this increase induces a phase change, sphere positions adjust significantly to fill the additional space more effectively, thereby raising ϕ. If the phase remains unchanged, only subtle shifts in sphere positions occur, which may not fully capitalize on the added space and thus lead to slight decreases in ϕ.

G. Table-tennis ball experiments

PMMA tube	No. 1	No. 2	No. 3	No. 4	No. 5
Minor axis (mm)	75.0 ± 0.2	78.3 ± 0.1	73.6 ± 0.2	81.2 ± 0.2	83.3
Major axis (mm)	76.3	80.2	74.2 ± 0.1	81.9 ± 0.2	83.9 ± 0.2

Table S2. Five elliptic PMMA tubes of different sizes.

To further understand the densest packings in elliptic cylinders, we devise macroscopic experiments to support the simulations. By replacing the elliptic cylinders and spheres in the simulation with transparent elliptic tubes and table-tennis balls, we can experimentally study the densest packing of balls inside the tubes. We purchase PMMA tubes with approximately elliptical cross sections and measure their dimensions to choose suitable tubes for the experiments. For the cross-sectional measurements, a digital vernier caliper is used to measure the inner major and inner minor axes of the section, and the average values and error ranges are obtained from repeated measurements. The accuracy of the caliper is ± 0.2 mm. The measurements are compared with the geometric ranges required by the phase diagram, and tubes with dimensions within the allowed tolerance are selected for the experiments. We employ five PMMA tubes of different sizes (see Table S2) and a sufficient number of table-tennis balls (diameter $40 - 40.1$mm). Using these five tubes and repeated packing experiments with table-tennis balls, we can reproduce the eight phases observed in the simulations.

Phases	Tubes	Experimental packing fraction	Theoretical packing fraction	Relative error (%)
Zigzag	No. 3	0.3660 – 0.3702	0.3718 – 0.3761	1.56
Helical phase I	No. 2	0.4113 – 0.4154	0.4324 – 0.4385	5.08
Helical phase II	No. 2	0.4121 – 0.4163	0.4261 – 0.4307	4.32
Double oscillated-chain ($s > 3$)	No. 1	0.3779 – 0.3832	—	—
Double oscillated-chain ($s = 3$)	No. 1	0.3825 – 0.3880	0.3906 – 0.3930	1.67
Double oscillated-chain ($s = 2$)	No. 2	0.4189 – 0.4231	0.4219 – 0.4283	0.96
Tilted achiral doublet	No. 4	0.4752 – 0.4805	—	—
Triple-chain	No. 5	0.4885 – 0.4934	—	—

Table S3. A comparison of the experimental and theoretical packing fractions of the eight phases.

Table S3 compares the experimental and theoretical packing fractions for the eight phases. Taking into account the tube dimensional error (Table S2), the packing fraction is reported as a range. In the experiments, we extract a segment of an elliptic tube, record its height and the number of table-tennis balls inside, and derive the packing fraction by dividing the total ball volume by the tube segment volume. To remove boundary effects, the height was measured from the center of the lowest ball to the center of the highest ball. For the theoretical packing fractions, the zigzag and the double oscillated-chain phases ($s = 3$ and $s = 2$) have short periods, so they can be found directly in the experiments and their exact theoretical solutions computed. However, one full period of helical phase I and helical phase II cannot be realized with the number of balls used in the experiments (this limitation also appears in simulations). Therefore, to match the experiments, the theoretical packing fractions of these two phases are computed with the triplet-sphere packing block method using the same number of balls as in the experiments (the height differences are computed using the same method as in the experiment, and periodic boundary conditions are not applied). No theoretical results currently exist for the double oscillated-chain ($s > 3$), the tilted achiral doublet, and the triple-chain, so values for these phases are omitted. Thus, we have observed all eight phases experimentally, measured their packing fractions, and compared them with theoretical values. From Table S3, the maximum discrepancy is no greater than 5.1%, indicating the results are in close agreement and further supporting the simulations.

H. More details of the simulation

We first explain how the simulation points are selected. In this study, the minor axis of the elliptic cylinder satisfies $D_b \in [1.60,\, 2.00]$. For the circular cylinder, the diameter ranges corresponding to the first three phases are zigzag ($D \in \left[1.60,\, 1+\sqrt{3}/2\right]$), single helix ($D \in \left(1+\sqrt{3}/2,\, 1+4\sqrt{3}/7\right]$), and double helix ($D \in \left(1+4\sqrt{3}/7,\, 2.00\right)$). When extending from circular to elliptic cylinders, we start from these three phases and select an equal number of simulation

points for each phase. Ten points are uniformly chosen within each phase range, avoiding too few points from any single phase due to direct uniform sampling. If we sampled points uniformly, most of the points would come from the zigzag structure of the circular cylinder, while fewer would come from its helical structures. In this work, however, we focus more on the structures that evolve from the helical phases of the circular cylinder, so we use the sampling method described above. The aspect ratio satisfies $D_a/D_b \in [1,\, 1.10]$. Sampling is uniform in eccentricity $e = \sqrt{1 - \left(\frac{D_b}{D_a}\right)^2}$, so that more points are taken near the circular cylindrical limit and fewer points away from it. Because in this study we aim to observe how the packing structure changes as the circular cylinder transforms into the elliptic cylinder, we need to pay more attention to the elliptic cylinders close to the circular cylindrical limit. Therefore, we choose more points at smaller aspect ratios. Starting from $e = 0.00$ ($D_a/D_b = 1.00$), points are taken with increments of 0.02 up to $e = 0.42$ ($D_a/D_b \approx 1.10$), yielding 22 points. The total number of simulation points is $3 \times 10 \times 22 = 660$.

One more point should be emphasized in the simulation process. For each $(D_a/D_b,\, D_b)$, we first restart the simulation following the steps described in the main text. We perform multiple runs and select the densest packing structure among them. In some cases, however, the obtained structure differs from the densest packing structure in its neighborhood, and the packing fractions differ greatly. In these cases, the simulation may not have reached the densest structure. To solve this problem, we take the neighboring structure as the initial structure and reduce the initial temperature of simulated annealing, so that the sphere positions are only slightly adjusted, and the most likely densest packing structure is obtained. These cases that require recalculation are mainly located near the boundaries between different phases. By combining these two methods, we aim to identify the densest packing structure for each $(D_a/D_b,\, D_b)$ to the greatest extent possible.

Supplementary References

[1] H.-K. Chan, Y. Wang, and H. Han, Densest helical structures of hard spheres in narrow confinement: An analytic derivation, AIP Adv. **9**, 1 (2019).
