# Supplementary material for: Chiral packings in cylinders are ultrasensitive to confinement deformation
Source: Nat Commun. 2026 Jul 16;17:6398. doi: 10.1038/s41467-026-74709-2 (PMC13376757; doi:10.1038/s41467-026-74709-2)
Supplement: Supplementary file 2 — Transparent Peer Review file [file 41467_2026_74709_MOESM2_ESM.pdf]

Peer Review File

Chiral Packings in Cylinders are Ultrasensitive to Confinement Deformation

Corresponding Author: Professor Yao Li

This file contains all reviewer reports in order by version, followed by all author rebuttals in order by version.

Version 0:

Reviewer comments:

Reviewer #1

(Remarks to the Author)
There is a well known sphere packing problem wherein spheres are packed inside a narrow circular cylinder, and chiral helical structures are often observed. However, these structures are not seen in model biological systems. The authors of this paper find that perturbing the circular cylinder into an elliptical cylinder allows for new packing types which match the biological data, and interestingly, these structures appear under very small perturbation, suggesting that the chiral helical structures are extremely sensitive to these perturbations.

Given the wide biological impact of the original papers that study packings in circular cyllinders and the fact that this elliptical perturbation is 1) analytically tractable, 2) biologically relevant, and 3) represents fundamentally new structures, I believe that it fits within the scope of Nature Communications. The paper is also written at a level which is appropriate for the wider audience that Nature Communications attracts. I have only a few suggestions which I believe will make this suitable for publication.

1. This may seem pedantic, but I think the language which calls these hard spheres may need light modification. Certainly, I agree that these are hard spheres once the packings are made and the energy is minimized such that they have no overlap (or their overlap is vanishingly small). I simply think that Eq. 1 and 2 should be refered to as soft contact potentials and that the interactions between spheres are taken initially to be soft until this minimization is complete. There has been a decent amount of work showing that the thermal and mechanical behavior of soft spheres under a countact potential and hard spheres is rather different.

2. It is implied in this work that the densest packing is also the most stable, and this has not been shown (or cited) explicitly. Stating clearly that this is the assumption will help to clarify things.

3. While it is clear to me why they choose the values of D_b that they use (because they align with the various regions of interest in Fig. 1), it may help to state at the outset which values they will be considering or to refer back to Fig. 1 each time a new value is chosen.

Reviewer #2

(Remarks to the Author)
This manuscript describes theory, computer simulations and a limited number of experiments concerning the arrangements of spheres in tubes of elliptical cross-section. Numerous studies have been published recently which identified the type of ordered packings that are obtained for cylindrical tubes. The current work shows that the deviation from this symmetry leads to a number of new phases, such as double oscillated chains or tilted achiral doublets. No confinement in nature will be perfectly elliptical, but the findings presented here explain why some structures that have been recorded for example in the zebrafish notochord differed from the previously reported structures. Deviation from circularity invites different arrangement. The work is a welcome and overdue extension to the current knowledge on sphere packings in cylinders.

The manuscript is well written and I am happy to recommend publication once the following queries are addressed.

Figure 1: Mention in the caption that the tube diameter is set to 1.

Phase diagram shown in figure 1: how many individual pairs (D_a/D_b, D_a) were probed for its construction?

Once a dense packing was obtained, were its coordinates used to probe in the neighbourhood of (D_a/D_b, D_a) or was the simulation started anew for different (D_a/D_b, D_a)? (Work by other authors points to the strong presence of hysteresis in this context.)

There is published data for the case D_a=D_b for the demarcation of phase boundaries. Is this data being reproduced with the methods described in the manuscript?

Experiments with ping-pong balls: How were the tubes of elliptical cross-section obtained? This would be worth mentioning in the main text.

p3: "predicts most of the simulations" --> "predicts most of the MC simulations"

p3, left column, last two sentences: "packing fraction increases with N and clearly exceeds the simultations. This implies that periodic BC with finite N affect the densest packings." Please discuss this a bit more here.

p4: left column, last paragraph: "theoretical densest packings" (dashed lines), where do these values originate from?

Figure 6: "for s=2 and s=3" --> "for segment lengths s=2 and s=3 (see Figure 1)"

page 6, first column, text starting with "For a circular cylinder ..." Why is this section so late in the manuscript. It makes connections with known work on cylinder packings and could be placed earlier to motivate the current work as it highlights the effect of eccentricity.

Version 1:

Reviewer comments:

Reviewer #1

(Remarks to the Author)
The authors have adequately addressed my previous concerns, and I would like to recommend the article for publication in its current form.

As a closing comment, I would like to echo and emphasize the statement made by the second reviewer:

"No confinement in nature will be perfectly elliptical, but the findings presented here explain why some structures that have been recorded for example in the zebrafish notochord differed from the previously reported structures. Deviation from circularity invites different arrangement."

This elliptical perturbation has revealed that the type of sphere packings seen in a circular tube are highly sensitive to perfect circularity, and are thus not seen in biological systems, which are far from perfect geometric objects. This first order perturbation has opened up a whole new zoo of possibilities, and I hope that it will inspire further investigations into the stability of each packing type to various biologically relevant perturbations.

Reviewer #2

(Remarks to the Author)
I am satisfied that the authors took my comments on board. I am happy to recommend publication of the revised manuscript.

Open Access This Peer Review File is licensed under a Creative Commons Attribution 4.0 International License, which permits use, sharing, adaptation, distribution and reproduction in any medium or format, as long as you give appropriate credit to the original author(s) and the source, provide a link to the Creative Commons license, and indicate if changes were made.
In cases where reviewers are anonymous, credit should be given to 'Anonymous Referee' and the source.
The images or other third party material in this Peer Review File are included in the article's Creative Commons license, unless indicated otherwise in a credit line to the material. If material is not included in the article's Creative Commons license and your intended use is not permitted by statutory regulation or exceeds the permitted use, you will need to obtain permission directly from the copyright holder.
To view a copy of this license, visit https://creativecommons.org/licenses/by/4.0/

Reviewer 1:

Comment 1:

This may seem pedantic, but I think the language which calls these hard spheres may need light modification. Certainly, I agree that these are hard spheres once the packings are made and the energy is minimized such that they have no overlap (or their overlap is vanishingly small). I simply think that Eq. 1 and 2 should be refered to as soft contact potentials and that the interactions between spheres are taken initially to be soft until this minimization is complete. There has been a decent amount of work showing that the thermal and mechanical behavior of soft spheres under a countact potential and hard spheres is rather different.

Reply:

Thank you for this helpful comment. We agree that until the densest packing is achieved, the spheres should not be regarded as hard spheres. They are hard spheres only when the interaction energy is zero, with no overlap between the spheres and between the spheres and the wall. We have revised the manuscript to make this point clearer to readers. Please refer to the red text on page 7 of the manuscript.

Comment 2:

It is implied in this work that the densest packing is also the most stable, and this has not been shown (or cited) explicitly. Stating clearly that this is the assumption will help to clarify things.

Reply:

Thank you for this helpful comment. This question can also be answered by the reply provided in Comment 1. When the spheres just separate from each other and from the wall, they also just become hard spheres, and the total interaction energy is then zero. This indicates that the obtained densest structure must be the most stable one. The revisions we made in reply to Comment 1 are sufficient for readers to understand this point.

Comment 3:

While it is clear to me why they choose the values of D_b that they use (because they align with the various regions of interest in Fig. 1), it may help to state at the outset which values they will be considering or to refer back to Fig. 1 each time a new value is chosen.

Reply:

Thank you for this helpful comment. We agree that referring to Fig. 1 when introducing new values of the minor axis D_b of the elliptic cylinder will help readers better understand. Please refer to the red text

on page 3: (the orange segment in the phase diagram of Fig. 1);

on page 5: (the yellow segment in the phase diagram of Fig. 1);

and on page 6: ($D_b = 1.878397$, the purple segment in the phase diagram of Fig. 1).

Reviewer 2:

Comment 1:

Figure 1: Mention in the caption that the tube diameter is set to 1.

Reply:

Thank you for this helpful comment. We actually set the hard-sphere diameter to $d = 1$. We have clarified this point in the manuscript, which should make it easier for readers to understand. Please refer to the red text in the caption of Fig. 1 of the manuscript.

Comment 2:

Phase diagram shown in figure 1: how many individual pairs $(D_a/D_b, D_a)$ were probed for its construction?

Reply:

Thank you for this helpful comment. We actually use individual pairs $(D_a/D_b, D_b)$. We selected 30 values within the minor-axis range of the elliptic cylinder and 22 values within the aspect-ratio range, resulting in 660 simulation points in total. However, the

procedure for selecting these points is complicated and has already been briefly described in the Simulation Process section of the Methods in the manuscript. We now discuss it in more detail in the Supplemental Material so that readers can understand how the simulation points were selected. Please refer to the first paragraph in Section H of the Supplemental Material.

Comment 3:

Once a dense packing was obtained, were its coordinates used to probe in the neighbourhood of $(D_a/D_b, D_a)$ or was the simulation started anew for different $(D_a/D_b, D_a)$? (Work by other authors points to the strong presence of hysteresis in this context.)

Reply:

Thank you for this helpful comment. Similarly, we actually use individual pairs $(D_a/D_b, D_b)$. For each different $(D_a/D_b, D_b)$, we first restart the simulation. However, when the obtained structure may not be the densest packing structure, we run the simulation again using the densest structure in the neighborhood as the initial structure. Thus, we use both methods to find the densest structure as far as possible. We have added a discussion of this part in the Supplemental Material, hoping to help readers understand how we search for the densest packing structure. Please refer to the second paragraph in Section H of the Supplemental Material.

Comment 4:

There is published data for the case $D_a = D_b$ for the demarcation of phase boundaries. Is this data being reproduced with the methods described in the manuscript?

Reply:

Thank you for this helpful comment. For the case $D_a = D_b$, the elliptic cylinder becomes a circular cylinder, and the triplet-sphere packing block method is still applicable. However, because twisted periodic boundary conditions were not used in the simulation, the simulation results did not reach the densest packing structure. In the figure below, $N \leq 25$ and $N \leq 6400$ represent the packing fractions of the

structures constructed by the triplet-sphere packing block method with sphere numbers $N \leq 25$ and $N \leq 6400$, respectively. These values match the packing fractions obtained from the simulation results and the theoretical densest helical structures. This indicates that the triplet-sphere packing block method is also applicable to the circular cylinder. We have added a discussion of this part in the Supplemental Material. Please refer to the red text in Section C of the Supplemental Material.

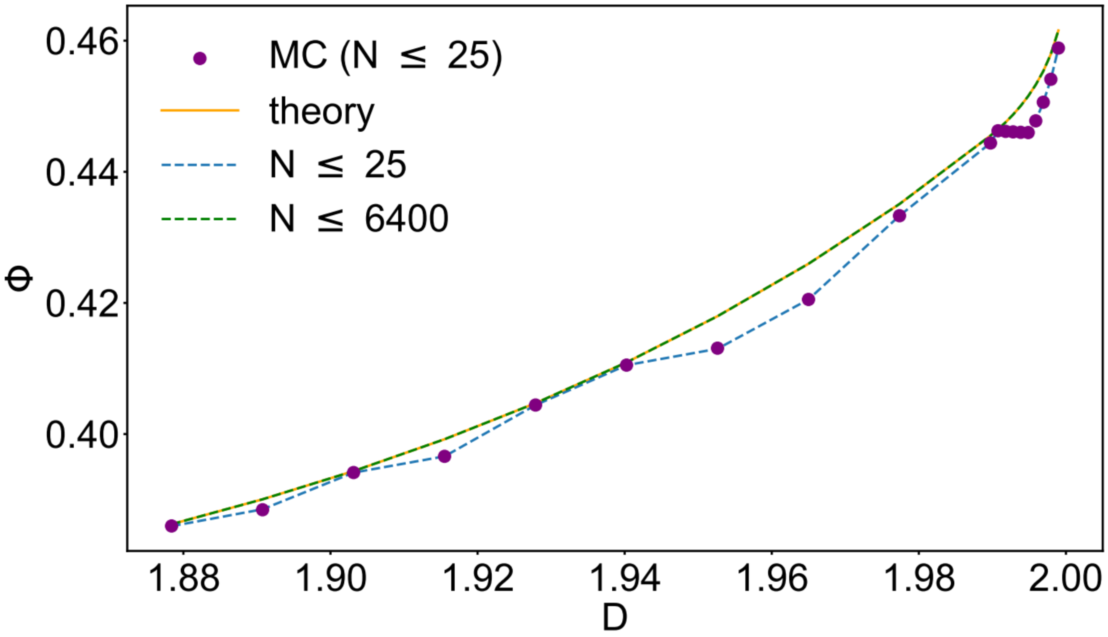

Figure 1: The points represent the MC simulation results for $N \leq 25$. The solid line represents the analytical expressions for the helical phases in the circular cylinder, as reported in previous studies, while the dashed lines show the results obtained by the triplet-sphere packing block method with $N \leq 25$ (blue dashed line) and $N \leq 6400$ (green dashed line).

Comment 5:

Experiments with ping-pong balls: How were the tubes of elliptical cross-section obtained? This would be worth mentioning in the main text.

Reply:

Thank you for this helpful comment. We first purchase some cylindrical PMMA tubes, but these tubes are difficult to process under our available conditions. We find, however, that these tubes already have a small eccentricity and satisfy our requirements. We therefore measure them with a vernier caliper and select several tubes with suitable dimensions for the experiments. We have provided a brief description of the tube

selection in the Supplemental Material. Please refer to the red text in Section G of the Supplemental Material.

Comment 6:

p3: "predicts most of the simulations" --> "predicts most of the MC simulations"

Reply:

Thank you for this helpful comment. We have revised the manuscript accordingly. Please refer to the red text on page 3: predicts most of the MC simulations.

Comment 7:

p3, left column, last two sentences: "packing fraction increases with N and clearly exceeds the simultations. This implies that periodic BC with finite N affect the densest packings." Please discuss this a bit more here.

Reply:

Thank you for this helpful comment. This problem indeed needs to be discussed in more detail. We have added a discussion of this issue in the manuscript to help readers better understand the effect of periodic boundary conditions with finite N. Please refer to the red text that spans the left and right columns on page 3 of the manuscript.

Comment 8:

p4: left column, last paragraph: "theoretical densest packings" (dashed lines), where do these values originate from?

Reply:

The "theoretical densest packings" are obtained using the triplet-sphere packing block method with $N \leq 6400$, where the effects of finite sphere number and periodic boundaries can be neglected, so that the densest structures can be reliably determined. The values indicated by the dashed lines A, B, C, and D in Fig. 3(a) are the packing fractions of the structures obtained by the triplet-sphere packing block method with $N \leq 25$. They are selected to discuss the differences between the simulation results and the densest packings. They correspond to the four structures shown in Fig. 5(a)–(d),

respectively, as noted in the caption of Fig. 3 of the manuscript. To help readers better understand this point, we have also revised the manuscript accordingly. Please refer to the red text that spans the left and right columns on page 4 of the manuscript, as well as the red text in the last paragraph of the right column on page 4.

Comment 9:

Figure 6: "for $s = 2$ and $s = 3$" --> "for segment lengths $s = 2$ and $s = 3$ (see Figure 1)"

Reply:

Thank you for this helpful comment. We have revised the manuscript accordingly. Please refer to the red text in the caption of Fig. 6 of the manuscript.

Comment 10:

page 6, first column, text starting with "For a circular cylinder ..." Why is this section so late in the manuscript. It makes connections with known work on cylinder packings and could be placed earlier to motivate the current work as it highlights the effect of eccentricity.

Reply:

Thank you for this helpful comment. We have moved the discussion of the zigzag structure to an earlier part of the manuscript, so that we can start with the simplest structure and better illustrate the effect of the elliptic cylinder on the packing structure. Please refer to the red text in the first paragraph of the left column on page 3 of the manuscript.
